# Modified duct-to-mucosa versus conventional pancreaticoenterostomy for pancreaticoduodenectomy: a retrospective cohort study based on propensity score matching analysis

**DOI:** 10.1186/s12957-018-1557-5

**Published:** 2019-01-05

**Authors:** Tianchong Wu, Yuehua Guo, Jiangang Bi, Shuwang Liu, Yusheng Guo, Shiyun Bao

**Affiliations:** Department of Hepatobiliary and Pancreatic Surgery, The Second Medical College, Shenzhen People’s Hospital, Jinan University, Shenzhen, 518020 Guangdong Province China

**Keywords:** Pancreaticoenterostomy, Postoperative pancreatic fistula, Pancreaticoduodenectomy

## Abstract

**Background:**

Clinically relevant postoperative pancreatic fistula (CR-POPF) remains the most common neopathy after pancreatoduodenectomy (PD). An ideal pancreaticoenterostomy (PE) which can effectively reduce the incidence of CR-POPF and its potential neopathy is needed. We aimed to assess the efficacy of our modified duct-to-mucosa PE in the PD.

**Method:**

From January 2011 to December 2017, 233 consecutive patients with PD were retrospectively included from Shenzhen People’s Hospital. After propensity score matching (PSM), there were 82 patients in both the modified duct-to-mucosa PE group (group A) and the conventional end-to-side inserting PE group (group B), respectively. The clinical course and the incidence of postoperative neopathy were compared between groups. Logistic regression method was utilized to analyze the association between PE approach and CR-POPF.

**Results:**

The PE time was shorter in group A (9.3 ± 1.8 min vs. 21.5 ± 2.8 min, *P* < 0.001). The group A had significantly lower incidence of severe neopathy (Clavien–Dindo grade > II) [7.3% (5/82) vs. 17.1% (14/82), *P* = 0.028] and incidence of CR-POPF [1.2% (1/82) vs. 19.5% (12/82), *P* < 0.001] than the group B. Our modified duct-to-mucosa PE technique was associated with a reduced risk for CR-POPF (OR, 0.11 [95% CI, 0.02–0.57]; *P* = 0.009) as compared with the conventional end-to-side inserting PE.

**Conclusion:**

Compared with conventional end-to-side inserting PE, our modified duct-to-mucosa PE technique can effectively reduce the incidences of postoperative neopathy and CR-POPF.

**Trial registration:**

Researchregistry3877. Registered 24 March 2018. Retrospectively registered.

## Background

Pancreaticoduodenectomy (PD) is still the first-line treatment for pancreatic head carcinoma, primary malignant duodenal tumors, and periampullary carcinoma [1]. At present, PD is a safe treatment in a majority of high-capacity surgical departments, with a mortality rate lower than 3–5% [2]. However, although the mortality rate is low, the rate of overall complications remains high (40–54%) [2–5]. It has been shown that 33% of postoperative patients have clinically relevant postoperative pancreatic fistula (CR-POPF) [3]. CR-POPF significantly impacts on intra-abdominal infection, reoperation rate, post-pancreatectomy hemorrhage, time of hospitalization, unplanned readmission rate, and mortality. Multiple risk factors are associated with CR-POPF including pancreas texture, pancreatic duct diameter, blood supply of pancreatic stump, and surgical technique [3].

The pancreaticoenterostomy (PE) technique is the crucial step in the PD operation [4]. Several pancreatic anastomosis techniques, including end-to-end invaginated PE, duct-to-mucosa PE, conventional end-to-side inserting PE, and pancreaticogastrostomy, have been proposed and used as an alternative option for pancreatic anastomosis. In spite of this, there is no unified understanding of the pancreatic anastomosis technique which can effectively reduce the occurrence of CR-POPF and its potential complications [5–9]. An ideal anastomosis technique for PE should be easy to perform in any pancreatic duct size and pancreatic texture and induce a low rate of CR-POPF [9]. In this study, we reported a simple and feasible PE technique with modified duct-to-mucosa anastomosis technique. Our modified duct-to-mucosa PE technique was named as single-layer continuous running suture technique and has been being performed since 2013 in our department. The aim of this study was to investigate the effect of our modified duct-to-mucosa PE technique for PD based on propensity score matching (PSM) study model.

## Methods

### Patients and data collection

The patients in an institution (Shenzhen People’s Hospital) receiving PD treatment for various lesions, including pancreatic cancer, bile duct cancer, and Vater’s papilla cancer, between January 2011 and December 2017 were included. In this study, the conventional end-to-side inserting PE was performed between 2011 and 2013, and the modified duct-to-mucosa PE was performed between 2013 and 2017. The inclusion criteria were as follows: (1) over 18 years of age, (2) tolerated to surgery, and (3) postoperative life expectancy of at least 3 months [9]. The exclusion criteria were as follows: (1) laparoscopic PDs and (2) required multiple organ resection. Data collection included clinical characteristics, operative findings, postoperative outcomes, and details of the postoperative complications. The data was reported according to the Strengthening the Reporting of Cohort Studies in Surgery (STROCSS) guidelines for cohort study [10].

All of the operations were completed by the same one surgeon Shiyun Bao, who had over 20 years of clinical experience in hepatobiliary and pancreatic surgery and independent experience of more than 200 cases of PD. In the patients with malignancy, D2 regional lymphadenectomy was performed. If the superior mesenteric vein or the portal vein were violated, the vessels were resected and reconstructed. The neck of the pancreas is cut by a scalpel. The detailed procedure of PE was described in the following section. The choledochojejunostomy and gastroenterostomy were finished by Child’s reconstruction model. Two abdominal drainage tubes (22Fr) were placed at peripancreatic and right subhepatic separately. The content of amylase in the drainage fluid was routinely measured at 3 days postoperation. When the CR-POPF or other anastomotic fistula did not occur, the drainage tube would be dismantled. Abdominal neopathy, such as CR-POPF, intra-abdominal infection, or chylous fistula, were treated with continuous intraperitoneal drainage therapy. However, when these treatments fail to control the complications, reoperation would be performed immediately before the patients had serious complications, such as septicemia or shock.

### Techniques of PE

#### The surgical technique for modified duct-to-mucosa PE

**Fig. 1:**
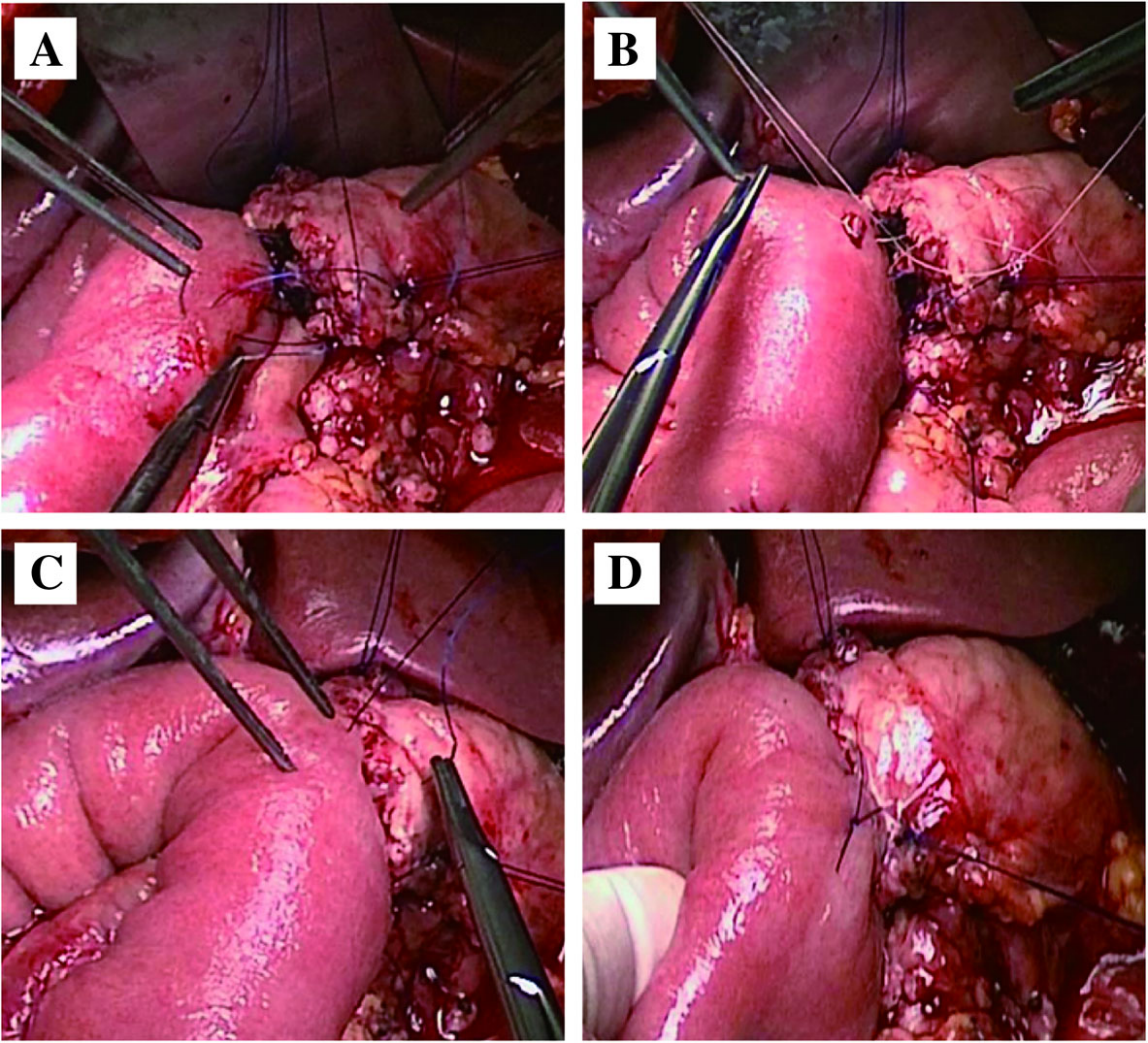
Surgical procedures for modified duct-to-mucosa pancreaticoenterostomy: **a** Single-layer continuous running suture in the posterior wall of the anastomotic stoma. **b** Discontinuously suture of the main pancreatic duct and the jejunum mucous membrane. **c** Single layer continuous running suture in the anterior wall of the anastomotic stoma. **d** Pancreaticoenterostomy was completed

The modified duct-to-mucosa PE was completed by an end-to-side anastomotic method. The posterior wall of anastomotic stoma was finished firstly with single-layer continuous running suture technique by 4-0 polypropylene suture (Prolene; Ethicon, Inc., NJ) while step by step to tighten the suture between the pancreas and seromuscular layer from upper margin of the pancreas to the lower margin (Fig. 1a). A stab incision in the corresponding jejunum mucosa was then made by an electric surgical knife. After that, the corresponding size of the pancreatic duct stenting tube (6–8 Fr) was used as an endoluminal stent between the main pancreatic duct and jejunal lumen. The main pancreatic duct and the jejunum mucous membrane incision were then discontinuously sutured by 6 (pancreatic duct diameter < 2 mm) to 8 (pancreatic duct diameter > 5 mm) stitches with 4-0 VICRYL sutures (Fig. 1b). The anterior wall of anastomotic stoma was performed also with single-layer continuous running suture technique using the other end of the same 4-0 polypropylene suture (Prolene; Ethicon, Inc., NJ) while step by step to tighten the suture between the pancreas and seromuscular layer from the upper margin of the pancreas to the lower margin (Fig. 1c). All stitches were set to 5-mm intervals (Fig. 1d).

#### The conventional end-to-side inserting PE

The conventional end-to-side inserting PE was carried out just as previously described [6, 11, 12]. In brief, the pancreatic stump was separated from the capsule of the pancreas and its length was not less than 2 cm. The pancreatic duct stenting tube (6–8 Fr) was used as an endoluminal stent in all cases. An incision was performed on the corresponding jejunum with the same diameter as the residual end of the pancreas. The stump of the pancreas was inserted into the incision of jejunum. Firstly, continuous suture was performed between the whole layer of jejunum and the pancreas by using 3-0 polypropylene suture (Prolene; Ethicon, Inc., NJ). Secondly, the pancreatic parenchyma and jejunal seromuscular were interrupted sutured by using 4-0 polypropylene suture (Prolene; Ethicon, Inc., NJ).

### PSM analysis

To investigate the relationship between the surgical selection and the clinical outcomes, the clinical characteristics which significantly differed in the before PSM cohort (in Table 1) were included in the PSM analysis [11] to eliminate the potentially confounding effect. In addition, the variables associated with the surgical options and the efficacy were also included in the PSM analysis. The variables included in the PSM analysis were performance status, gender, age, American Society of Anesthesiologists class (ASA), body mass index (BMI), previous laparotomy, jaundice, cholangitis, weight loss, tumor size, hemoglobin (HB), albumin (ALB), preoperative bile duct drainage, comorbidities, pancreatic duct diameter, and pancreatic texture. The selected variables were subjected to probit regression to produce the continuous propensity score (ranging 0 to 1). The nearest neighbor of 1:1 was matched between the patients in group A and group B, by which the miscellaneous factors in the patiens selection can be reduced, and the matched patients were screened for subsequent steps of analysis.

**Table 1:** Clinical characteristics of patients in the two groups before and after PSM

Variables	Group (before PSM)			Group (after PSM)		
	A (*n* = 124)	B (*n* = 109)	*P* value	A (*n* = 82)	B (*n* = 82)	*P* value
Performance status (0:1:2)	119:5:0	105:4:0	0.580	78:4:0	80:2:0	0.682
Gender (female/male)	50:74	58:51	0.033	37:45	39:43	0.754
Age (years), *n* (%)						
< 45	10 (8.1)	2 (1.8)	0.037	8 (9.8)	1 (1.2)	0.696
45–55	38 (30.6)	28 (25.7)		23 (28.0)	27 (32.9)	
56–65	50 (40.3)	61 (56.0)		34 (41.5)	43 (52.4)	
> 65	26 (21.0)	18 (16.5)		17 (20.7)	11 (13.4)	
ASA, *n* (%)						
I	120 (96.8)	105 (96.3)	0.566	78 (95.1)	82 (97.6)	0.405
II	4 (3.2)	4 (3.7)		4 (4.9)	2 (2.4)	
III	0	0		0	0	
IV.V	0	0		0	0	
BMI (kg/m^2^), *n* (%)						
< 24	90 (54.2)	76 (45.8)	0.037	64 (78.0)	60 (73.2)	0.502
24–28	33 (60.0)	22 (40.0)		17 (20.7)	21 (25.6)	
> 28	11 (91.7)	1 (8.3)		1 (1.2)	1 (1.2)	
Previous laparotomy, *n* (%)	5 (4.0)	14 (12.8)	0.014	5 (6.1)	7 (8.5)	0.549
Jaundice, *n* (%)	50 (40.3)	66 (60.6)	0.002	47 (57.3)	44 (53.7)	0.637
Cholangitis, *n* (%)	33 (26.6)	44 (40.4)	0.026	29 (35.4)	30 (36.6)	0.871
Weight loss, *n* (%)	38 (44.2)	48 (55.8)	0.035	29 (35.4)	28 (34.1)	0.870
Tumor size, *n* (%)						
< 2 cm	44 (35.5)	29 (26.6)	0.026	25 (30.5)	22 (26.8)	0.869
2–4 cm	56 (45.2)	68 (62.4)		47 (57.3)	49 (59.8)	
> 4 cm	24 (19.4)	12 (11.0)		10 (12.2)	11 (13.4)	
HB (g/L), *n* (%)						
≧ 10	111 (89.5)	91 (83.5)	0.176	74 (90.2)	71 (86.6)	0.464
< 10	13 (10.5)	18 (16.5)		8 (9.8)	11 (13.4)	
ALB (g/L), *n* (%)						
≧ 35	90 (72.6)	63 (57.8)	0.018	54 (65.9)	51 (62.2)	0.625
< 35	34 (27.4)	46 (42.2)		28 (34.1)	31 (37.8)	
Preoperative bile duct drainage, *n* (%)	30 (24.2)	40 (36.7)	0.038	27 (32.9)	26 (31.7)	0.867
Comorbidities, *n* (%)						
Diabetes	18 (14.5)	28 (25.7)	0.033	13 (15.9)	16 (19.5)	0.539
Respiratory disease	11 (8.9)	12 (11.0)	0.585	6 (7.3)	6 (7.3)	> 0.999
Ischemic heart disease	10 (8.1)	8 (7.3)	0.836	7 (8.5)	6 (7.3)	0.773
Hypertension	40 (32.3)	30 (27.5)	0.431	22 (26.8)	23 (28.0)	0.861
Pancreatic duct diameter, *n* (%)						
< 3 mm	19 (15.3)	30 (27.5)	0.023	14 (17.1)	16 (19.5)	0.686
≧ 3 mm	105 (84.7)	79 (72.5)		68 (82.9)	66 (80.5)	
Pancreas texture, *n* (%)						
Soft	37 (29.8)	35 (32.1)	0.708	22 (26.8)	23 (28.0)	0.861
Firm	87 (70.2)	74 (67.9)		60 (73.2)	59 (72.0)	

*PSM* propensity score matching

### Definition of postoperative complications

Postoperative complications score were determined according to the Clavien–Dindo classification [13]. CR-POPF was classified on the basis of the ISGPF (International Study Group of Pancreatic Fistula) 2016 criteria [3]. Biliary fistula was defined as the abdominal drainage containing bile components which the total bilirubin level was higher than the normal level at 3 days postoperation. Chylous fistula was defined as the milky white appearance or triglyceride content of peritoneal drainage fluid exceeding 110 mg/dl. Delayed gastric emptying was defined as having to use a gastric tube for 7 or more days after the operation or the diagnosis was confirmed by digestive tract angiography. In-hospital mortality was defined as the death of the patient during hospitalization or within 30 days after the operation.

### Statistical analysis

Continuous data were indicated as mean ± standard deviation (SD) while categorical data were presented as number and percentage. Differences in categorical data between the two groups were determined by *chi-square* test or Fisher’s exact test (if any expected value lower than 5 was found), and the differences between continuous data were tested by Mann–Whitney *U* test or Student’s independent *t* test. The factors related to CR-POPF were determined by the logistic regression model. The statistical analysis was performed by SPSS 22.0 software for Windows (SPSS Inc., Chicago, IL, USA). *P* < 0.05 was deemed to be statistically significant, two-tailed.

## Results

### Clinical characteristics of patients before and after PSM

Before PSM, there were 233 patients who met the standard of research participated in the study. Of these, 124 patients (74 males and 50 females) received our modified duct-to-mucosa PE, and 109 sufferers (51 males and 58 females) underwent the conventional end-to-side inserting PE. The two groups had comparable performance status, ASA, levels of HB, respiratory disease, ischemic heart disease, hypertension, and pancreas texture (all *P* > 0.05). However, there were significant differences in gender, age, BMI, previous laparotomy, jaundice, cholangitis, weight loss, tumor size, levels of ALB, preoperative bile duct drainage, diabetes, and pancreatic duct diameter between the two groups (all *P* < 0.05). After PSM, there was effectively balance in the factors with dominant differences before propensity matching. The PSM was performed successfully to generate 82 pairs of matched patients from the two groups. There was no significant difference in all parameters between the groups (Table 1).

### Operative findings and postoperative outcomes in the PSM model

The operative time of group A was shorter than that of group B (214.6 + 28.5 min vs. 229.8 + 26.5 min, *P* < 0.001), because of the significant shorter PE time in group A (9.3 ± 1.8 min vs. 21.5 ± 2.8 min, *P* < 0.001). There were no significant differences in blood loss, delayed gastric emptying, and ICU transfer between the two groups (*P* > 0.05). Intra-operative blood transfusion of group A was significantly less than that of group B (*P* = 0.025). In group A, the postoperative hospital stay was significantly shortened (*P* = 0.008), which was similar to the patients before PSM (group A vs. B, 15.6 ± 3.2 vs. 18.2 ± 5.6; *P* < 0.001). According to the final pathological diagnosis, there was no significant difference between the groups (*P* > 0.05) (Table 2).

**Table 2:** Operative findings and postoperative outcomes in PSM model

Operative time (min)	Group A (*n* = 82)	Group B (*n* = 82)	*P* value
	214.6 ± 28.5	229.8 ± 26.5	< 0.001
Pancreaticoenterostomy time (min)	9.3 ± 1.8	21.5 ± 2.8	< 0.001
Blood loss (ml)	314.4 ± 147.6	349.9 ± 132.4	0.107
Intra-operative blood transfusion (ml), *n* (%)			
None	44 (53.7)	27 (32.9)	0.025
≦ 600	17 (20.7)	27 (32.9)	
> 600	21 (25.6)	28 (34.1)	
Delayed gastric emptying, *n* (%)	4 (4.9)	9 (11.0)	0.148
ICU transfer, *n* (%)	4 (4.9)	6 (7.3)	0.514
Postoperative hospital stay (days)	15.1 ± 2.8	17.74 ± 4.9	0.008
Final diagnosis, *n* (%)			
Pancreatic cancer	42 (51.2)	33 (40.2)	0.158
Bile duct cancer	7 (8.5)	7 (8.5)	> 0.999
Intraductal papillary mucinous neoplasm	8 (9.8)	9 (11.0)	0.798
Vater’s papilla cancer	5 (6.1)	4 (4.9)	0.732
Neuroendocrine tumor	4 (4.9)	6 (7.3)	0.514
Metastasis from other organs	6 (7.3)	5 (6.1)	0.755
GIST	1 (1.2)	3 (3.7)	0.620
Chronic pancreatitis	1 (1.2)	6 (7.3)	0.053
Solid pseudopapillary tumor	6 (7.3)	6 (7.3)	> 0.999
Duodenal cancer	2 (2.4)	3 (3.7)	0.650

*PSM* propensity score matching

### Postoperative complications in the PSM model

Table 3 reported the details of postoperative complications on the basis of the Clavien–Dindo scale. In the group B, there were 2 patients died during hospitalization and 1 patient died within 90 days after the operation. Among them, 2 patients succumbed to multiple organ failure, and the other one died of post-pancreatectomy hemorrhage. The postoperative complications (Clavien–Dindo classification grade I–II), such as in post-pancreatectomy hemorrhage, GI anastomotic fistula, chylous fistula, septicemia, gastrointestinal hemorrhage, and readmission, were not significantly different between the two groups (all *P* > 0.05). However, group A was superior to group B in the occurrence of severe complications (*P* = 0.028). In group A, only 5 patients had a severe complication at the Clavien–Dindo III level. By contrast, there were 14 patients with severe complications in group B, including 8 cases of Clavien–Dindo grade III, 5 cases of Clavien–Dindo grade IV, and 1 case of Clavien–Dindo grade V. In the unmatched cohort (*n* = 233), there were more mild to moderate (grade I–II) cases in group B (*n* = 29, 26.6%) as compared with group A (*n* = 10, 8.1%), and more severe (grade III–V) cases [20.2% vs. 0.8%, both *P* < 0.01). The incidence of biochemical leakage in group A was less than that in group B, but there is no apparent difference between the two groups. (18.3% vs. 25.6%; *P* = 0.258). A total of 13 patients had CR-POPF. In group A, the incidence of CR-POPF was significantly less than in group B (1.2% vs. 19.5%, *P* < 0.001). Furthermore, the incidence of grade B CR-POPF of group A was less than that of group B (1.2% vs. 12.2%, *P* = 0.005). Nevertheless, the incidences of grade C CR-POPF were not significantly different between the two groups (0.0% vs. 2.4%, *P* = 0.497).

**Table 3:** Details of the postoperative complications in PSM model

	Group A (*n* = 82)	Group B (*n* = 82)	*P* value
Biochemical leak, *n* (%)	15 (18.3)	21 (25.6)	0.258
CR-POPF, *n* (%)	1 (1.2)	12 (19.5)	< 0.001
Grade B	1 (1.2)	10 (12.2)	0.005
Grade C	0	2 (2.4)	0.497
Post-pancreatectomy hemorrhage, *n* (%)	0	4 (4.9)	0.043
GI anastomotic fistula, *n* (%)	0	2 (2.4)	0.497
Intra-abdominal infection, *n* (%)	3 (3.7)	10 (12.2)	0.043
Chylous fistula, n (%)	3 (3.7)	1 (1.2)	0.620
Septicemia, *n* (%)	1 (1.2)	2 (2.4)	0.556
Gastrointestinal hemorrhage, *n* (%)	3 (3.7)	1 (1.2)	0.620
Biliary fistula, *n* (%)	2 (2.4)	3 (3.7)	0.650
Organ failure, *n* (%)	1 (1.2)	2 (2.4)	0.500
In-hospital mortality	0	2 (2.4)	0.497
Readmission (< 1 month)	1 (1.2)	2 (2.4)	0.500
Mortality, 90 days	0	1 (1.2)	0.316
Clavien–Dindo classification			
Mild–moderate (grade I–II), *n* (%)	18 (22.0)	29 (35.4)	0.057
Severe (grade III–V), *n* (%)	5 (7.3)	14 (17.1)	0.028
Postoperative complications (total), *n* (%)	23 (28.0)	43 (52.4)	0.001

*PSM* propensity score matching

### Risk factors for pancreatic fistula

After adjustment for the potential confounding factors, our modified duct-to-mucosa PE was associated with a lower risk of CR-POPF (OR, 0.11 [95% CI, 0.02–0.57]; *P* = 0.009) as compared with the conventional end-to-side inserting PE. Furthermore, the BMI 24 kg/m^2^–28 kg/m^2^ (OR, 11.89 [95% CI, 2.14–65.94]; *P* = 0.005) or BMI > 28 kg/m^2^ (OR, 49.39 [95% CI, 5.20–469.19]; *P* < 0.001), diabetes (OR, 10.54 [95% CI, 2.55–43.61]; *P* = 0.001), and pancreatic duct diameter (OR, 6.72 [95% CI, 1.33–33.88]; *P* = 0.021) were identified as independent risk factors for CR-POPF in both univariate and multivariate analyses. The smaller the diameter of the pancreatic duct, the greater possibility of CR-POPF would be. For patients with a diameter of pancreatic duct less than 3 mm, the risk of CR-POPF was 8 of 41 (16.3%) as compared with 10 of 174 (5.4%) in those with diameter ≧ 3 mm (*P* = 0.029). Patients with soft pancreas texture were more likely to have CR-POPF than those with firm pancreas texture (*P* = 0.018). There were only 8 (5.0%) patients with firm pancreas texture who had CR-POPF compared with 10 (13.9%) patients who had a soft pancreas, respectively. (Table 4).

**Table 4:** Logistic regression model for CR-POPF occurrence in 233 unmatched patients

Variable	Univariate			Multivariate	
	With CR-POPF, *n* (%)	Without CR-POPF, *n* (%)	*P* value	Relative risk (95% CI)	*P* value
PE approach			< 0.001		0.009
End-to-side	17 (15.6)	92 (84.4)		1	
Duct-to-mucosa	1 (0.8)	123 (99.2)		0.11 (0.02–0.57)	
BMI (kg/m^2^)			< 0.001		< 0.001
< 24	5 (3.0)	161 (97.0)		Ref.	–
24–28	6 (10.9)	49 (89.1)		11.89 (2.14–65.94)	0.005
> 28	7 (58.3)	5 (41.7)		49.39 (5.20–469.19)	< 0.001
Diabetes			< 0.001		0.001
No	6 (3.2)	181 (96.8)		1	
Yes	12 (26.1)	34 (73.9)		10.54 (2.55–43.61)	
Final diagnosis			0.016		0.324
Pancreatic cancer	6 (4.3)	134 (95.7)		1	
Other pathologic findings	12 (12.9)	81 (87.1)		2.24 (0.45–11.10)	
Pancreas texture			0.018		0.480
Firm	8 (5.0)	153 (95.0)		1	
Soft	10 (13.9)	62 (86.1)		1.81 (0.35–9.45)	
Pancreatic duct diameter			0.029		0.021
≧ 3 mm	10 (5.4)	174 (94.6)		1	
< 3 mm	8 (16.3)	41 (83.7)		6.72 (1.33–33.88)	
Neoplastic			0.175		0.865
No	2 (20.0)	8 (80.0)		1	
Yes	16 (7.2)	207 (92.8)		0.73 (0.02–26.69)	

The logistic regression model of PSM cohorts was also been analyzed. As shown in Table 5, in the univariate results, the PE approach, diabetes, and neoplastic variables were significant and had a similar distribution to the cohorts before PSM. However, only diabetes and neoplastic variables were significant in the multivariate model, and it seems that patients with diabetes and without neoplastic variables were more likely to have CR-POPF (Table 5).

**Table 5:** Logistic regression model for CR-POPF occurrence in 164 matched patients

Variable	Univariate			Multivariate	
	With CR-POPF, *n* (%)	Without CR-POPF, *n* (%)	*P* value	Relative risk (95% CI)	*P* value
PE approach			0.034		0.156
End-to-side	8 (9.8)	74 (90.2)		1	
Duct-to-mucosa	1 (1.2)	81 (98.8)		0.19 (0.02–1.88)	
BMI (kg/m^2^)			0.090		0.030
< 24	4 (3.2)	120 (96.8)		1	–
24–28	5 (13.2)	33 (86.8)		15.75 (2.04–121.40)	0.008
> 28	0 (0.0)	2 (100.0)		0.00 (0.00–0.00)	1.000
Diabetes			< 0.001		0.005
No	3 (2.2)	132 (97.8)		1	
Yes	6 (20.7)	23 (79.3)		14.01 (2.20–89.19)	
Final diagnosis			0.936		0.791
Pancreatic cancer	4 (5.3)	71 (94.7)		1	
Other pathologic findings	5 (5.6)	84 (94.4)		0.71 (0.06–8.99)	
Pancreas texture			0.707		0.817
Firm	6 (5.0)	113 (95.0)		1	
Soft	3 (6.7)	42 (93.3)		0.75 (0.07–8.38)	
Pancreatic duct diameter			0.213		0.114
≧ 3 mm	6 (4.5)	128 (95.5)		1	
< 3 mm	3 (10.0)	27 (90.0)		5.39 (0.67–43.63)	
Neoplastic					0.021
No	2 (28.6)	5 (71.4)	0.049	1	
Yes	7 (4.5)	150 (95.5)		0.03 (0.00–0.59)	

## Discussion

This study compared perioperative effects of our modified duct-to-mucosa PE and conventional end-to-side inserting PE for PD. Despite the retrospective design of this study, the patient’s factors were controlled by PSM analysis and all operations were performed by the same surgeon. The clinical characteristics of patients and surgical conditions for PE in the two groups, such as the pancreatic texture and diameter of the pancreatic duct, were well balanced by using the PSM method. Therefore, this study still can provide evidence to assess the efficacy of our modified duct-to-mucosa PE techniques. PE is the committed step in the PD. Although some random studies and meta-analysis have been carried out, there is no evidence or guidelines on how to establish the best PE after PD [2–4, 6–8, 14, 15]. In recent 20 years, despite a significant decline in surgical mortality, the incidence of postoperative complications remains high (about 50%), and the incidence of serious complications of PD is relatively unchanged [16]. The postoperative complications rate is a significant difference based on the ISGPF diagnostic criteria [3]. In a first-class Chinese hospital (the annual case of PD is about 200), Liu et al. have reported that the overall incidence of pancreatic fistula was 64%, of which 33% was CR-POPF [17]. In this study, the incidence of CR-POPF in group B was 19.5%, suggesting that our overall surgical skill level of conventional PE techniques from 2011 to 2013 was comparable with that of the large Chinese hospital. However, our incidence of CR-POPF was still higher than that of other reports (11.2%) [11].

Of our paired matched patients, biochemical leakage did not significantly differ among the regimens (*P* = 0.258). However, the incidence of CR-POPF in group A (1.2%) was significantly lower than that reported by McMillan etal. [11]. CR-POPF is the main cause of morbidity and mortality after PD [3]. This could also explain the lower postoperative complications (*P* = 0.001) in group A (*P* = 0.001) and a shorter postoperative stay (*P* = 0.008). There are numerous risk factors associated with the occurrence of pancreatic leakage: (1) systemic factors, including gerontism, aurigo, and cacotrophy; (2) surgical factors, including intraoperative hemorrhage, lengthening of operation time, operation mode, pancreatic texture, the diameter of the pancreatic duct, and PE technique [18]. Among them, PE technique is the most crucial factor. To prevent pancreatic fistula, different types of surgical techniques to treat the residual end of the pancreas have been proposed [1–4, 6–8, 14, 15]. However, the efficacy of these techniques remains controversial, and no one is better than other methods [18]. Bartoli et al. [19] reported that postoperative pancreatic fistula occurred in end-to-side invagination PE was 26%, which was significantly higher than those of the duct-to-mucosa PE (16%). Peng et al. [20] believed that Peng’s binding PE was a safer and more efficient method. They also considered that pancreatic surgeons should master more than one type of PE techniques to deal with a different pancreatic stump. By contrast, Casadei et al. [21] reported that Peng’s binding PE cannot effectively reduce the occurrence of pancreatic fistula in the European population.

At present, there are two main PE methods: the “end-to-side inserting” PE technique and the “duct-to-mucosa” PE technique [12, 18]. The end-to-side inserting PE is a relatively simple method, and the necrotic tissue and secretions can be drained into the intestines in time. Nevertheless, the transverse section of the pancreas is directly exposed to the intestinal cavity, which may lead to erosion or even life-threatening bleeding [22]. Furthermore, cicatricial stenosis may develop in the pancreatic duct outlet [7, 9, 23]. The routine “duct-to-mucosa” PE has a good tissue fusion, but is a complicated operation. The technical requirement is high, and the surgeon needs a certain clinical experience. There is a definite space between the pancreatic stump and the intestinal wall, and pancreatic juice or exudant of pancreatic stump may accumulate which can result in rupture of the anastomosis. When pancreatic duct diameter is less than 3 mm, the success rate of the “duct-to-mucosa” PE is reduced [18, 24].

To reduce the incidence rate of CR-POPF, we developed a modified “duct-to-mucosa” PE with single-layer continuous running suture. We found that only 15 patients (18.3%) had a biochemical leak and 1 patient (1.2%) had grade B-type CR-POPF in the PSM model. No case of grade C-type CR-POPF was found in group A. Furthermore, compared with group B, the morbidity and mortality associated with pancreatic fistula were greatly reduced in group A. The severe postoperative complication (Clavien–Dindo grade > II) of group A was significantly lower than that of group B (*P* = 0.028). The reason for the significant reduction of CR-POPF in the group A could be attributed to the following reasons: (1) No sufficient free anatomy is required for the pancreatic stump, thereby the risk of pancreatic injury is reduced. (2) There are fewer stitches and pinholes made in the pancreatic stump by means of monolayer continuous suture, which causes less damage to the pancreas. (3) The anterior and posterior wall of the pancreatic stump was continuously sutured with the jejunum seromuscular layer, which can prevent leakage of succus pancreaticus from the pancreatic stump causing a pancreatic fistula. (4) The thickness of the pancreatic stump is no more a key factor, and the anastomosis is more in line with the physiological conditions. (5) With the continuous suture between the pancreatic stump and the jejunum seromuscular layer, the surface of the pancreatic stump was not easily eroding and bleeding. In our PSM series, only 4 cases of post-pancreatectomy hemorrhages were found in group B (*P* = 0.043). (6) Our modified duct-to-mucosa PE is technically uncomplicated, easy to operate. Compared with the conventional end-to-side inserting PE, performing a PE using our modified technique only needed 9.3 ± 1.8 min, which was greatly shorter than that of the PD (*P* < 0.001).

In addition, the risk factors for pancreatic fistula have been reported [5–9, 16, 25]. However, associated risk factors for CR-POPF after PD based on the ISGPF 2016 criteria have not yet been clarified [3, 18]. As suggested by Babyak [26], the number of independent variables included in the multivariate logistic regression models should be determined based on the number of events occurrences in the dependent variable. One independent variable could be included every 10–20 events occurrences in the dependent variable. In this study, the number of CR-POPF occurrences was 18, suggesting that only two independent variables could be included in the model. In this study, the variables controlled in the logistic regression model were chosen by clinical experiences and theoretical knowledge, which should keep the model stable simultaneously. Finally, we chose the following 6 variables controlled in the multivariate logistic regression model: PE approach, BMI, diabetes, final diagnosis, pancreas texture, and pancreatic duct diameter. The effect of our modified duct-to-mucosa PE on the occurrence of CR-POPF was tested in both the unmatched cohort (233 patients) and the matched cohort (164 patients) by logistic regression modeling. After the adjustment of the potential confounding factors, the PE approach was a significant factor in both univariate/multivariate results before PSM and in the univariate results after PSM. However, the PE approach was not significant in the multivariate results after PSM (*P* = 0.114), which may be attributed to the reduced sample size of after PSM cohort. However, the consistency among these all four models still indicated that PE approach was a potential factor associated with the occurrence of CR-POPF. On the other hand, the ISGPF recommends that an assessment of the risk factors of pancreatic fistula should take into account the pathological diagnosis [3]. We found that it was a predictor based on the single or multiple variable analyses. Especially, the patients with pancreatic cancer were less likely to have pancreatic fistula compared with those with other lesions (OR, 2.31 [95% CI, 1.47–6.05]; *P* = 0.009). Logistic regression analysis of our unmatched cohort also showed that CR-POPF was closely related to the factors including BMI, diabetes, pancreas texture, and pancreatic duct diameter. There are several limitations to this study. First, it is limited by its retrospective nature; however, the application of PSM analysis could effectively reduce the treatment selection bias. In addition, as a single-center study, there were some internal biases. The results from a single surgeon may be not representative enough. Furthermore, in this study, the conventional PE technique was performed between 2011 and 2013, and the modified duct-to-mucosa PE was performed between 2013 and 2017. It should be pointed out that the different surgical methods in the separated period may influence the outcome of surgical safety.

## Conclusions

In conclusion, this study compared the efficacy of our modified duct-to-mucosa PE and the conventional end-to-side inserting PE in the PD by using PSM analysis. The results show that our modified duct-to-mucosa PE technique is a feasible surgical procedure for PD, which has a good efficacy in terms of CR-POPF rate and other major perioperative outcomes as compared with the conventional end-to-side inserting PE, which may be a better surgical technique for the patients undergoing PD.

## Abbreviations

ALB: Albumin
ASA: American Society of Anesthesiologists class
BMI: Body mass index
CI: Confidence intervals
CR-POPF: Clinically relevant postoperative pancreatic fistula
HB: Hemoglobin
ISGPF: International Study Group of Pancreatic Fistula
PD: Pancreatoduodenectomy
PE: Pancreaticoenterostomy
PSM: Propensity score matching
STROCSS: Strengthening the Reporting of Cohort Studies in Surgery
